# Substance use among Palestinian youth in the West Bank, Palestine: a qualitative investigation

**DOI:** 10.1186/s12889-016-3472-4

**Published:** 2016-08-17

**Authors:** Salwa G. Massad, Mohammed Shaheen, Rita Karam, Ryan Brown, Peter Glick, Sebastian Linnemay, Umaiyeh Khammash

**Affiliations:** 1 Palestinian National Institute of Public Health, Ramallah, West Bank Palestine; 2 Al Quds University, Abu Dis, Palestine; 3 Rand Corporation, Santa Monica, USA; 4 United Nations Relief and Works Agency for Palestine Refugees in the Near East (UNRWA), Jerusalem, Palestine; 5 Juzoor for Health and Social Development, Al-Arkan St, Al-Bireh, West Bank Palestine

**Keywords:** Youth, Alcohol, Drugs, Palestine

## Abstract

**Background:**

Youth health risk behaviors, including substance use (psychoactive substances including alcohol and illicit drugs), have been the subject of relatively limited study to date in Middle Eastern countries. This study provides insights into the perceived prevalence and patterns of alcohol and drug use among Palestinian youth.

**Methods:**

The study was based on ten focus groups and 17 individual interviews with youth aged 16–24 years (*n* = 83), collected as part of the formative phase of a cross-sectional, population representative study of risk taking behaviors among Palestinian youth in the West Bank in 2012. Qualitative analysis was used to code detailed notes of focus groups and interviews.

**Results:**

Most participants reported that substance use exists, even in socially conservative communities. Almost all participants agreed that alcohol consumption is common and that alcohol is easily available. The top alcoholic drinks referred to by the study participants were vodka, whisky, beer, and wine. Most participants claimed that they drink alcohol to cope with stress, for fun, out of curiosity, to challenge society, and due to the influence of the media. Participants were familiar with illicit drugs and knew of youth who engaged in drug use: marijuana, cocaine, and heroin were mentioned most frequently. Study participants believed that youth use drugs as a result of stress, the Israeli occupation, inadequate parental control, lack of awareness, unhappiness, curiosity, and for entertainment. Many participants were unaware of any local institutions to support youth with substance use problems. Others expressed their distrust of any such institution as they assumed them to be inefficient, profit-driven, and posing the risk of potential breaches of confidentiality.

**Conclusions:**

Although this study uses a purposive sample, the results suggest that substance use exists among Palestinian youth. Risk behaviors are a concern given inadequate youth-friendly counseling services and the strong cultural constraints on open discussion or education about the impact of high risk behaviors. These barriers to treatment and counseling can exacerbate the health and social consequences of alcohol abuse and illicit drug use.

## Background

Political violence is a health threat to adolescents; the experience of violence can have long-term emotional effects and may foster risky behaviors, including substance use [1–4]. Youth in Palestine and their families are profoundly affected by the Israeli occupation and repeated exposure to political violence. It affects all aspects of everyday lives, including unemployment and economic hardship, school closings, frequent humiliation and harassment, and travel restrictions. The pathways to high-risk behaviors are complex and include biological mechanisms whereby stress adversely affects cognitive function and decision making capacity, including avoidance or engagement in high-risk behaviors as a means of restoring self-pride and self-respect [3].

Extensive research has been conducted to identify factors associated with drug use among children and adolescents. The risk factors identified include exposure to drugs, socio-economic status, quality of parenting, peer pressure, school and neighborhood influences, biological/inherent predisposition towards drug addiction [8], antisocial behavior, risky sexual practices, and academic failure [9]. Protective factors include academic success and participation in volunteer activities [9]. However, the potential impact of specific risk and protective factors can vary with age; family has a stronger influence on a younger child, while peer group influence may be a more significant risk factor for an adolescent [10].

In addition to an increased risk of chronic diseases at an older age [5], substance use in adolescents is associated with health risks such as depression, motor vehicle crashes, risky sexual behaviors, and suicidal behavior [6]. Regular use of psychoactive substances such as alcohol and illicit drugs can lead to dependence, and users may persist with this habit despite the harmful consequences [7].

In Palestine, there are indications that drug use among youth is relatively high and is increasing despite the religious, legal, and cultural constraints. Drug use is a growing concern, especially in Palestinian East Jerusalem where the prevalence of alcohol and drug use is particularly high [11]. In addition to easy access to drugs or alcohol from Israel, several factors may contribute to the high levels of risk behaviors in East Jerusalem: economic stagnation, poor social services, significant social and political tensions, and the inability of Palestinian law enforcement authorities to police this area. As a result, drugs classified as illegal in Palestine are widely traded in Jerusalem and drug dealers are not prosecuted [12]. Drug use among young people has been described as a “heroin plague” and East Jerusalem as a “safe haven for dealers and users” [12]. UN estimates from official sources indicate that there were around 10,000 drug users in the West Bank and Gaza Strip in 2007 (of a total population of 3.8 million), and about 15,000 in East Jerusalem [11] (of a total population of around 362,000 [13]).

A recent study of the prevalence of HIV and HIV-related risk taking behaviors among 82 Palestinian injecting drug users (IDU) in East Jerusalem, showed that more than 25 % of the study participants shared injecting equipment in the week preceding the survey. Add to that, only 34 % of IDUs reported using a condom during the most recent sexual intercourse [14], potentially setting the stage for an HIV epidemic within this sub-population and beyond it. These studies highlight the need for further research to understand substance use among Palestinian youth.

Several studies of children and adolescents in Palestine documented links between self-reported experiences of political violence and outcomes such as PTSD, poor mental health, and externalizing symptoms such as violent aggression [15–18]. However, little is known about how prolonged hardship and experiences of conflict at different levels (individual, family, and community) influence the propensity of young people in Palestine to engage in various risky behaviors or how these impacts vary by location or by demographic factors. Therefore, the specific aim of this qualitative research is to provide insights into the perceived prevalence of substance use among Palestinian youth, patterns and correlates, and the perceived quality and effectiveness of health services to help youth with substance use problems. An understanding of the patterns and causes of youth health risk behaviors will enable policymakers to develop and target appropriate prevention programs [19].

## Methods

As part of a larger quantitative study aimed at a systematic examination of the prevalence of health risk behaviors among Palestinian youth in the West Bank and to understand the determinants of these behaviors, we conducted 10 focus groups and 17 semi-structured interviews with Palestinian youth. This paper presents results from the study’s formative phase pertaining to youths’ perceptions of the prevalence of substance use and the settings in which such behaviors occur. In individual interviews, but not in focus groups, youth were also asked to discuss their own behaviors.

### Recruitment and sample

We utilized a combination of purposive and convenience sampling techniques to select a sample balanced across age, gender, region, residency (villages, refugee camps, towns, and cities), and to ensure adequate representation from employed, unemployed, out of school, in school and college students. We first selected communities from the southern, middle including East Jerusalem, and northern regions of the West Bank–including refugee camps and rural areas in some of those communities. Communities included Bethlehem, Hebron, Jericho, East Jerusalem, Kufr Aqab, Nablus, Ramallah, Shufat, Tulkarm, and Qalqylia. Recruitment was conducted by various organizations, including the Sharek Youth Organization, Juzoor for Health and Social Development, and the United Nations Relief and Works Agency for Palestine Refugees in the Near East.

Five focus groups of young women and five focus groups of young men were conducted, with six to nine participants in each group. A total of 17 young adults (eight females and nine males) participated in individual interviews to explore in-depth issues related to their own behavior: nine of them were recruited from the focus groups. A total of 83 participants (42 males and 41 females) participated in the study. Table 1 supplies a demographic description of the study sample, approximately half of whom were female, 51 participants were students, and 12 were in employment. With the exception of one young woman, all participants were single. Of the 83 participants, 26 were from East Jerusalem.

**Table 1:** Demographic characteristics of participants (*N* = 83)

Age		
Median		20
Range		16–24
	N	%
Males	42	50.6
Geographical distribution		
- North of West Bank	21	25.3
- Middle of West Bank	18	21.7
- South of West Bank	18	21.7
- East Jerusalem	26	31.3
Type of residency		
- Urban	30	36.2
- Rural	26	31.3
- Camp	27	32.5
Highest level of education		
- Less than high school	21	25.3
- High school	10	12.0
- College	31	37.4
- University	21	25.3
Employment status		
- Employed	12	14.4
- Unemployed	20	24.2
- Student	51	61.4

### Focus group and interview procedures

The focus groups and individual interview protocols explored the perceptions of young adults about the prevalence of health risk behaviors among their peers. Key questions included the perceived prevalence of substance use in Palestine; sub-groups of youth who engage in substance use; the most common types of substance use; sources of alcohol and drugs; settings for use (alone, with group, party..); the reasons youth engage in substance use; perceived gender and geographical variances in substance use; factors contributing to substance use; the perceived consequences of engaging in substance use; and the counseling and treatment services available to Palestinian youth affected by alcohol and drug abuse. In addition, key questions were presented to inform the quantitative study. These included a question on the most effective and acceptable survey method for use with youth, whether a face-to-face interview, a self-administered survey, an online survey, an interview via cell phone, or a computer-assisted survey. Other questions related to the setting: an interview in a private room in the home or outside the home, and perceived ways to make young people more comfortable and open to answering sensitive questions.

The interviewers also asked respondents about their own engagement in substance use. Both protocols were semi-structured to ensure that key questions were addressed and to permit comparisons across individuals and groups, while at the same time providing the interviewer with the freedom to follow up on unanticipated topics. Interviewer gender was matched with the gender of the focus group or interviewee and all interviews were conducted in Arabic. The focus group interviews were audio recorded after obtaining the participants’ consent. Separate focus groups were conducted for youth aged 15–17 and 18–24 years. Due to the fact that the individual interviews addressed sensitive personal behavior, these were not taped and the interviewer instead took detailed written notes. The RAND Corporation, Santa Monica, CA and Al-Quds University, Jerusalem, Palestine, approved the study protocol for ethical consideration in January 2012. Oral informed consent and assent were obtained from participants and the parents of those below 18 years of age.

#### Qualitative Analysis

All notes were translated from Arabic into English. A sample of the translated interviews was reviewed against the Arabic version by research team members to ensure accuracy. Qualitative analysis was conducted on the English version of the notes using NVIVO Version 9. Initial formal coding broke the data down into themes and sub-themes. The major themes were substance use and sexual activity. This paper focuses on substance use as sexual activity was discussed in a separate paper [20].

In accordance with accepted protocols for rigorous qualitative coding [21], each of these codes specified inclusion and exclusion criteria for the coding of excerpts, typical and atypical exemplars, and “close but no” examples. Within each of the formal codes referred to above, sub-codes were further developed to indicate discussion of sub-groups, locations, personal engagement in behaviors and the like.

Data were coded by one of the three researchers based in Palestine and two coders located in the US, who reviewed and validated the coding. Any coding differences were discussed and resolved. The sub-coded text was then summarized into bullet points with quotations and the frequency of each sub-code reported by the number of focus groups and individual interviews. Eight codes and 87 sub-codes resulted from the analysis for drugs, and 8 codes and 47 sub-codes resulted from the analysis for alcohol.

## Results

Many participants indicated that alcohol and drug use are prevalent, although alcohol to a greater extent. Substance use was perceived to be more prevalent among males and in East Jerusalem.

### Alcohol

#### Patterns and perceived prevalence of alcohol use

Many participants indicated that alcohol use was widespread to an extent that it could be considered “normal” in many areas of the West Bank and was easily available. In some cities, alcohol is served at weddings or parties and offered in dorms. Close to half the sample interviewed individually admitted to drinking alcohol. The top alcoholic drinks referred to by study participants were vodka, whisky, beer, and wine. Other types of alcohol used included arak, champagne, cocktails, and energy drinks mixed with vodka.

“It has become normal in every place.” [21 year old woman, East Jerusalem, focus group]

“In weddings, people drink a lot; some even faint from excessive drinking.” [24 year old woman, West Bank city, interview]

Many participants described young men as particularly heavy users of alcohol compared with young women, and single youth drink more than those who are married. No differences in alcohol use were perceived by educational level, student status, or employment. According to many study participants, young single men and women go out on Thursday nights (Friday is a weekend day) to drink, even in conservative communities.

“In parties, young men get drunk and dance in a funny way.” [23 year old woman, West Bank city, interview]

“We drink moderately, we do not abuse alcohol so that we will not get drunk and our families notice and find out that we drink.” [19 year old women, West Bank city, interview]

“Thursdays’ nightlife is like a prayer (*it is a must)*.” [A woman over the age of 19, West Bank camp, focus group]

According to all study participants, alcohol consumption in cities is higher than in villages. The city is more open; drinking in the city is easier and alcohol is available in shops, especially in Ramallah and Bethlehem. Several youth described Ramallah as the location where the most drinking occurs. This was followed by locations near Israel or Israeli settlements, and in refugee camps.

Alcohol consumption occurs in a wide variety of locations. Youth described obtaining alcohol most frequently from public establishments such as bars or restaurants, followed by friends or other acquaintances who purchased it mainly from Ramallah, Bethlehem or Israel. Some study participants stated that, although alcoholic drinks may not be visible on store shelves in some conservative cities, well-known stores that sell alcohol exist. A few study participants said that some people smuggle alcohol from Israel to the West Bank.

“There are used clothing suppliers who bring clothes from Israel. They smuggle beverages between the clothes into Palestinian Authority areas.” [A man over the age of 19, West Bank village, focus group]

Many participants said that several coffee shops in Ramallah offer alcohol at night. People who go for drinks are seen as the “elite or high class society” who speak in English.

“After 9:00 pm, the coffee shops in Ramallah turn into bars and everybody speaks English…” [21 year old woman, East Jerusalem, focus group]

“I drink with friends in private settings every other week, usually on Thursdays or Fridays. I drink with my friends in their houses when the parents are away; I do not drink in restaurants or coffee shops. But when I go to Jordan I drink in discos and pubs.” [19 year old woman, West Bank city, interview]

The next most popular setting for alcohol consumption was “undercover” or concealed consumption in public locations such as dorms, universities, and even cars.

“Some mix the alcoholic drink in the XL can or in a juice can and drink it in the club so the people around them will not know that they are drinking.” [18 year old man, West Bank city, interview]

“There were young people with me at university who used to drink in a secret way. They put the alcohol into a coca cola or a juice can and used to drink all day.” [19 year old man, West Bank camp, interview]

### Illicit Drug Use

#### Perceived prevalence and patterns of drug use

Most participants perceived drug use among youth in Palestinian society to be prevalent.

“Yes, drugs are increasing …drug abuse takes place in any location, at evening parties. It is easier to get than a pack of cigarettes.” [17 year old man, East Jerusalem, focus group]

Some even believe that drugs are affordable and cheaper than alcoholic drinks.

“At first, drug dealers sell them for a ridiculously cheap amount: only $2.5.” [A woman over the age of 18, East Jerusalem, focus group]

“Alcohol costs about $13 to $18 per person, whereas you could buy drugs for you and your friends for $13. That could make five or seven cigarettes; each person could roll them as they want, then they could take a puff or two.” [24 year old woman, East Jerusalem, interview]

Although both focus group participants and those interviewed individually referred to many types of drugs used by Palestinian youth, hashish/marijuana was the most popular drug. The second most frequently mentioned drug was cocaine, and heroin was the third most popular drug. Other types of drugs referred to included ecstasy, trip pills and synthetic LSD. Participants also mentioned the following youth groups that are susceptible to taking drugs: the very young, those subjected to abuse, those lacking education and/or jobs, those working in Israel (with access to drugs), youth living in urban areas and in camps, and young men more than young women. Participants emphasized that males tend to have more freedom to go out than females and are monitored less closely by their parents, thus having more opportunities of exposure to drugs.

“Sure, there are differences; the percentage of male users exceeds that of females. Guys can stay out for extended periods of time and are free to go anywhere.” [24 year old woman, West Bank city, focus group]

“I used ecstasy. It made me hallucinate and I used to see all the people looking like animals to me. I was always in a good mood. I used to take a pill or two in a week, either with my friends or when I went out at night.” [22 year old man, West Bank city, interview]

“Many young people in East Jerusalem secretly use ecstasy; I used it once in a party. I fell unconscious 1 hour after using it.” [24 year old man, East Jerusalem, interview]

Most people indicated that drugs come mainly from Israel; some even bring marijuana seeds from Israel and plant it around their houses.

“The main sources are Israeli. Israel gives drugs to collaborators, who distribute it to young Palestinians.” [23 year old woman, East Jerusalem, focus group]

“The majority of drugs come from Israel. I know guys from the West Bank who work in Israel and got seeds to sell to others in the West Bank.” [A woman over the age of 20, West Bank city, focus group]

Many stated that drugs are easily accessible; they access drugs through friends, drug dealers, and also prepare it at home. Youth engage in drug use in various locations. Many participants indicated that youth tend to use drugs in groups and with other friends, and to a lesser extent on their own.

“It is easier to get than a pack of cigarettes…” [A man over 19 years of age, East Jerusalem, focus group]

“It is easier to buy drugs in Jerusalem than in the West Bank; one third of drug dealers are found in the old city of Jerusalem.” [A man over the age of 20, East Jerusalem, focus group]

“Drugs are easily circulated everywhere, even in schools: in the street, in certain supermarkets, and in the Nescafe, cappuccino and chocolate stalls in Jerusalem. The man who sells falafel in our neighborhood also sells drugs.” [A man below the age of 20, East Jerusalem, focus group]

“Some people put spices or a drug on the water pipe and it acts as a drug.” [A 23 year old woman, East Jerusalem, focus group]

“The experience begins with a group of friends, and then the individual starts to use drugs alone.” [A woman over the age of 20, West Bank camp, focus group]

The most common places where youth use drugs are at parties, weddings, nightclubs, university dorms, abandoned houses, orchards, parties, private homes, and in empty areas (mountains and fields). Some reported that youth use drugs in zones (known as Area C) where there is a presence by neither the Palestinian nor the Israeli authorities. Drug use and trade are high in these areas.

“In my university days, I used to meet girls who would use drugs in the form of pills in the rest rooms.” [24 year old woman, West Bank city, focus group]

“Youth use drugs in abandoned old houses, in public rest rooms, and on trips.” [19 year old woman, West Bank camp, interview]

“They use drugs on the outskirts of Jerusalem in refugee camps.” [A woman over the age of 18, West Bank village, focus group]

“Sometimes I use drugs in the university dorm and on university field trips. I also used them in parties and once in a wedding; the people there wanted to do us a favor.” [24 year old man, Jerusalem, interview]

“Drugs are more common in Jerusalem than in the rest of the West Bank. In our area, there are many parks and forests. Nobody enters the area in the evening because it is known that drug users are gathered there at this time.” [21 year old woman, Jerusalem, focus group]

### Perceived causes of alcohol and drug use among Palestinian youth

Most participants claimed that youth drink alcohol to cope with stress, for fun, out of curiosity, to challenge the culture and society, and due to the influence of the media.

“Alcohol may affect my health, but it is better to die this way rather than to die with many worries in a heart attack. In either case, I am dead, so I may as well have fun and forget my worries.” [22 year old man, West Bank city, interview]

“Everyone in the camp drinks: both children and men. They drink to feel better and have fun.” [19 year old woman, West Bank camp, interview]

“No one who drinks is unaware of the health hazards, but forbidden fruit is sweet.” [A man over the age of 19, Jerusalem, focus group]

“I know about alcohol from watching Turkish [TV] series; they show that whoever drinks has prestige.” [A woman over the age of 19, West Bank city, focus group]

Just as with alcohol use, study participants believed that youth use drugs to cope with stress, for entertainment (to pass the time), and out of curiosity.

“Too many worries force youth to use drugs.” [A man under the age of 20, West Bank village, focus group]

“Young men take drugs because they want to get high; they want to feel good for a while, and there is nothing else to make them feel good.” [19 year old woman, West Bank city, interview]

Other perceived causes of illicit drug use were cited as the Israeli occupation, which encourages drug use by Palestinian youth, inadequate parental control and lack of awareness or feelings of unhappiness.

“Yes, drugs are increasing and are promoted by the occupation.” [A man over the age of 20, East Jerusalem, focus group]

“The Israeli occupation helps the spread of drugs to a large extent and drugs are available in significant quantities.” [A man below the age of 20, West Bank city, focus group]

“Drug addicts in Jerusalem receive an allowance from the Israeli National Insurance as long as they are on drugs. It is easier to take an allowance from the National Insurance than to work.” [A man over the age of 19, East Jerusalem, focus group]

“Young people are lost; they face neglect, indifference and lack of awareness.” [23 year old woman, East Jerusalem, focus group]

“Neglect by parents and the need to fill the time because nobody cares lead to drug use.” [A woman over the age of 19, West Bank, focus group]

### Access to rehabilitation and counseling services

According to study participants, Palestinian youth rarely receive any institutional support to help them deal with alcohol or drug use problems. Many participants did not know of any local institutions that offered support to youth with substance use problems. Others expressed their distrust of any institution and assumed them to be ineffective, profit-driven and prone to breaches of confidentiality. Another perceived barrier to seeking help with substance use is the social stigma.

“Palestinian youth often fail to get any support.” [A woman over the age of 19, West Bank city, focus group]

“There are institutions, but they are not reliable; they inform parents about the subject in hand.” [A woman over the age of 19, West Bank village, focus group]

“There are no specialized bodies. Even if they do exist, they carry out projects and leave.” [A woman over the age of 18, Jerusalem, focus group]

“Institutions in Jerusalem are weak and ineffective.” [A man over the age of 19, Jerusalem, focus group]

“Even if there are parties that provide assistance, addicts refrain from seeking help for their situation because they feel ashamed.” [A woman over the age of 18, Jerusalem, focus group]

## Discussion

Based on feedback from study participants, drinking takes place frequently. This finding is consistent with previous studies in other Arab countries [22]. Illicit drug use was also perceived to be prevalent, although less common than alcohol. In a previous study among adolescents aged 13–16 years from five countries, lifetime cannabis use was 2.3 % in Algeria, 3.7 % in Morocco, and 3.9 % in Palestine [23].

There were variances in substance use by gender and by residency. In line with previous studies, this study indicates that young males tend to drink and use illicit drugs more than females [23]. The gender difference may possibly be attributed to social norms that tend to stigmatize substance use in females more than in males. Also, risky behavior is more common among young males than among young females [23]. Many youth from rural areas visit cities to engage in substance use, maybe to escape the eyes of their communities, or because illegal substances and alcohol can be more easily obtained in the city and there is less stigma associated with drug use.

The Israeli occupation was cited most frequently as the cause of illicit drug use in Palestine. It is a widely held belief among Palestinians that Israel encourages substance use among Palestinians, especially youth, to destroy their future. This belief is supported by the fact that the Israeli police makes little effort to halt the drug supply to Arabs. The regular dealers can be seen in broad daylight day after day [12]. According to a previous study in Jerusalem, the military occupation and related violence, political instability, and economic hardship are likely to continue to generate conditions conducive to drug abuse in Palestine [14].

Stress was also one of the main causes cited for substance use. There is evidence of a high prevalence of depression and hopelessness among Palestinian youth and younger adolescents [24]. It is estimated that more than 30 % of Palestinian youth experience symptoms of post- traumatic stress disorder (PTSD) [15]. Evidence from previous studies suggests that experiences of community-level violence and of personal trauma increase the likelihood that young people will engage in risky behaviors, including smoking and drug use [4].

Another reported cause of drug use was to pass free time. In 2012, 26 % of youth aged 20–24 years in the West Bank were unemployed [25]. In addition to political violence, unemployment is also considered a risk factor for drug use [26].

There was consensus among study participants that services for youth are inadequate in Palestine and youth rarely receive any institutional support to help them deal with alcohol or drug use problems, a view supported in the existing literature [27, 28]. This may be explained by the strong cultural constraints on open discussion or education about the harmful effects of alcohol use and drug addiction [29]. A number of programs exist to tackle different aspects of the drug problem, but these are limited in scope and need to be intensified [28].

To the best of our knowledge, this is the first qualitative study of the health risk behaviors of young Palestinians. It has the standard limitation of a non-probability sample, namely that the youth interviewed may not be representative of Palestinian youth overall. The findings suggest that substance use may be more common than is currently assumed. Unemployment, poverty, persistent economic hardship, availability of modern media and the internet, and our unique political context where youth are exposed to both chronic and acute violence, may counteract the influence of conservative values and contribute to what appears to be prevalent risk behavior [10, 14]. While we cannot exclude the possibility that participants may have overstated or understated drug use, this possibility is unlikely to be the case in focus groups discussing young people in general, or for participants who are of the opinion that substance use exists even in conservative communities.

Thus, our findings suggest the need for policy responses, comprehensive interventions and preventive programs targeting youth of both genders. Outreach and education programs on alcohol and drug prevention for Palestinian youth are relatively undeveloped, as is the case in the region generally. Our findings provide guidance as to where such programs are most needed, starting in schools. Early intervention is vital to change a child’s life pathway away from risk taking behaviors [10]. Programs should make particular efforts to engage male youth, but should not ignore young females, who also engage in these behaviors, albeit to a lesser extent. Urban youth and those in camps (many of which are essentially low-income urban neighborhoods) are perceived to be at greater risk for these behaviors and this should be reflected in outreach efforts.

## Conclusions

Substance use in Palestine may be more common than is currently assumed despite the conservative social context. Risk behaviors are a concern in Palestine given the inadequate youth-friendly services and the strong cultural constraints on open discussion or education about high risk behaviors. These barriers to treatment and counseling can exacerbate the health and social consequences of alcohol abuse and illicit drug use.
